# Comparative study of the growth, stress status and reproductive capabilities of four wild-type zebrafish (*Danio rerio*) lines

**DOI:** 10.1186/s40659-024-00549-3

**Published:** 2024-09-19

**Authors:** Céline Chevalier, Clémence Denis, Sid-Ahmed Nedjar, Yannick Ledoré, Frédéric Silvestre, Bérénice Schaerlinger, Sylvain Milla

**Affiliations:** 1 University of Lorraine, INRAE, L2A, 54 000 Nancy, France; 2 Institute of Life, Earth and Environment (ILEE), University of Namur, URBE, 5000 Namur, Belgium

**Keywords:** Zebrafish, Reproduction, Growth, Stress

## Abstract

**Background:**

Zebrafish are widely used in various research fields and to fulfil the diverse research needs, numerous zebrafish lines are available, each with a unique domestication background, potentially resulting in intraspecies differences in specific biological functions. Few studies have compared multiple zebrafish lines under identical conditions to investigate both inter- and intra-line variability related to different functions. However, such variability could pose a challenge for the reproducibility of results in studies utilising zebrafish, particularly when the line used is not clearly specified. This study assessed growth, stress status (cortisol, serotonin) and reproductive capabilities (maturity, fecundity, fertilisation rate, sperm quality) of four commonly used wild-type zebrafish lines (AB, SJD, TU, WIK) using standardized protocols.

**Results:**

The stress markers levels were found to be similar across the lines, indicating that the endocrine stress status is robust to diverse domestication histories. Variations were observed in the growth and reproductive parameters. The lines exhibited differences in the timing of puberty (86 dpf for AB and SJD lines vs. 107 dpf for the WIK line) despite achieving similar sizes, suggesting that there are line-specific variations in the induction of maturation. Additionally, the AB line demonstrated higher sperm quality than did the other lines and higher fecundity and fertilization rates than did the SJD line. The AB line also exhibiting a smaller adult size but a heavier brain relative to its body weight.

**Conclusion:**

These findings emphasize the importance of line selection for zebrafish research, indicating that researchers should consider line-specific traits to ensure the biological relevance and reproducibility of the results.

**Supplementary Information:**

The online version contains supplementary material available at 10.1186/s40659-024-00549-3.

## Background

The use of animal models has always been crucial to improving our understanding of biological processes. For all types of animal models used, a wide variety of lines are available for researchers to choose from, depending on the questions they intend to address. Among mammal models, specifically rodents, a dozen rat lines are routinely used [1]. Natural variations can exist among these populations, and some studies have highlighted the differential features of each population [1–4]. These lines differ in their morphology, behavioural responses and metabolism, which means that some lines are more suited to specific research fields than others [1]. As these lines differ from one another, they may also exhibit variability in their responses, leading to differences in the interpretation of results. This is particularly concerning when two populations from the same line and source but maintained at different sites display behavioural and neurochemical differences [3]. Such variations between lines have also been demonstrated in aquatic models, including zebrafish (*Danio rerio*). In this species, among the approximately thirty wild-type (WT) lines that exist or have existed, fewer than ten, including the AB, SJD, TU or WIK [5], are commonly used in laboratories [6]. A WT fish line can be defined as a closed breeding population that harbours no defined phenotypic mutation [7]. However, uncontrolled induced variations can exist between WT lines. Recently, Padovani et al*.* demonstrated that the AB line had higher mortality than the TU line in an intestinal inflammation induced model, suggesting physiological differences between the two populations in terms of the immune response [8]. Another study showed that the AB line exhibited stronger activation of the corticotropic axis after stressor exposure than did the TL line, leading to the selection of the former line for subsequent stress experiments [9]. Furthermore, marked genetic differentiation between the lines [10–12] supports the notion that marked phenotypic divergence could also occur.

The dissimilarities observed among the different lines may be attributed to their domestication, which is a process that modifies both genotypes and phenotypes, including in terms of physiology and behaviour, across the generations produced in captivity [13]. Domestication can be defined as a new evolutionary context in which humans select and control the environment of the domesticated population and can select for certain phenotypes of wild species [13, 14]. However, importantly, all characteristics are interrelated, and the selection of one trait can have consequences for the others [14]. This is why each line of WT zebrafish may have a unique genetic background resulting from the particular domestication process to which the line was subjected, with factors including (1) the choice of the founder wild broodstock and their origins; (2) the capacity of the founder individuals to acclimate, which can induce early selection; (3) the conditions of the first reproduction in captivity (e.g., pair or mass reproduction); (4) the progressive adaptation to a specific captive environment from generation to generation and (5) the potential genetic selection to which the line is potentially subjected [13]. A wide variety of parameters are impacted by domestication, as reviewed by Milla et al*.* [13], and growth, response to stress and reproduction appear to be the main functions affected.

The precise history of domestication for each line is of great importance in its establishment, but there is still much grey area regarding the exact history of each line, even if some information is available. The AB line was originally derived from two populations acquired in the early 1970s at different times from two different pet stores in Oregon (USA) [6, 15]. Over the course of approximately 70 generations [15], haploid offspring were obtained by crossing unselected males with females screened for healthy and embryos with a good appearance [6] to reduce the transmission of lethal embryonic mutations [5, 6]. The current AB line was produced in the early 1990s and entailed crossing six different stocks of diploid offspring stemming from distinct haploid females [15]. Currently, the stock is maintained through the breeding of mass reproduction groups [7, 15]. The TU line was derived from a German pet store stock purchased in 1994 and was inbred for several generations [15, 16]. Currently, the line is managed in Tübingen (Germany) [5, 15] and was used for the Zebrafish Sequencing Project of the Sanger Institute to assemble and annotate the zebrafish genome in the early 2000s [17]. The founder population underwent inbreeding and selection to minimize the transmission of lethal embryonic mutations, resulting in a more uniform line [18]. However, this may have led to more uncontrolled phenotypical changes than occurred in other lines that did not undergo such selection. This is particularly the case if the TU line is compared to the WIK line, which is much more polymorphic [19]. The latter originated from a single pair of wild-caught fish in 1997 in India [6, 19]. First described as the WIK11 line, the progeny were screened by Rauch et al. who discovered that this line had a probability exceeding 90% of being free of embryonic and larval lethality [19]. An additional example of the history of a WT zebrafish line is seen in the SJD (Steve Johnson Darjeeling) line, whose roots are traced to the offspring of a full Darjeeling wild-type sibship [6, 20] that was collected in 1987 in India and that in 1988 had its first-generation offspring sent to Oregon (USA), where it has been maintained by inbreeding [6, 20]. This represents a clonal line that was established through successive rounds of early pressure parthenogenesis [20], and it is more polymorphic than the AB, EKK, TL, TU and WIK lines [10, 21]. In the past, the Johnson laboratory was engaged in the selective breeding of these fish line to preserve their diversity [6].

We can hypothesise that the initial crossing event and the way each line was maintained influenced its genetic features and adaptation to laboratory conditions across generations. Given the rapid development of zebrafish use in research [22, 23], it is crucial to investigate potential phenotypic variation among the lines. A better understanding of the intraspecific differences among populations reared under the same conditions could also guide future line selection for specific scientific purposes. Currently, there is a lack of information on comparisons of multiple parameters belonging to different functions, such as reproduction, morphology, and stress markers, among several zebrafish lines reared under identical conditions. The aim of this study was to compare key parameters related to growth, stress response and reproduction among commonly used WT lines (AB, SJD, TU, and WIK) to detect potential differences and commonalities among them.

## Materials and methods

### Experimental animals and rearing conditions

All the experiment was conducted according to the European directive 2010/63/UE. Four lines were purchased from the European Zebrafish Resource Center (EZRC) (Karlsruhe, Germany): AB (WT embryo #1175), SJD (WT embryo #30,607), TU (WT embryo #1173) and WIK (WT embryo #1171). The embryos were transferred the same day to the Aquaculture Experimental Platform (registration number for animal experimentation C5454718) belonging to the L2A laboratory at the University of Lorraine (Nancy, France). For each line, embryos arrived at the same age (2 days post-fertilisation, dpf) in 4 groups, each one coming from several batches derived from an unknown number of parents with unknown degrees of genetic relationship. The groups remained unchanged throughout the experiment, setting 4 replicates per line that we considered as independent. The embryos were kept in embryo medium (5 mM NaCl, 0.17 mM KCl, 0.33 mM CaCl, 0.33 mM MgSO_4_, 7.2 < pH < 7.4) in Petri dishes placed in an incubator at 28.5 °C until hatching (72 h post-fertilisation, hpf). After hatching, the larvae were placed in 8 L aquariums (17 cm × 28 cm × 18 cm, L × W × H) with recirculating water (a renewal rate of 10% per day with osmosis water) at 27 ± 1 °C, with a density of 6 fish/L. A 10-h dark and 14-h light photoperiod was applied, with 30 min of dawn and dusk and 300 lx of light intensity at the surface water. Over the course of the experiment, the temperature, photoperiod and light intensity were controlled automatically by central technical management. Three times a week, the pH (7.5 ± 0.5), conductivity (850 ± 50 µS/cm) and ammonia and nitrite nitrogen levels were measured and remained below 0.1 mg/L. Mortality was monitored daily but remained anecdotal and equivalent between lines, which did not significantly affect density.

The dry food Gemma Micro ZF (Skretting, Fontaine-lès-Vervins, France; proteins 59%; lipids 14%; fibre 0,2%; minerals 14%; phosphorus 1,3%; calcium 1,5%; sodium 0,7%; vitamin A 23000 UI/kg; vitamin D3 2800 UI/kg; vitamin C 1000 mg/kg; vitamin E 400 mg/kg) was distributed ad libitum in different granulometry depending on the age of the fish. ZF75 was used 3 times per day between 5 and 15 dpf. ZF150 was used twice a day between 15 and 90 dpf, followed by ZF300 once per day until the end of the experiment. From 5 dpf until the end of the experiment, *Artemia nauplii* (EG > 225) was given as a supplement once per day.

### Experimental design

**Fig. 1:**
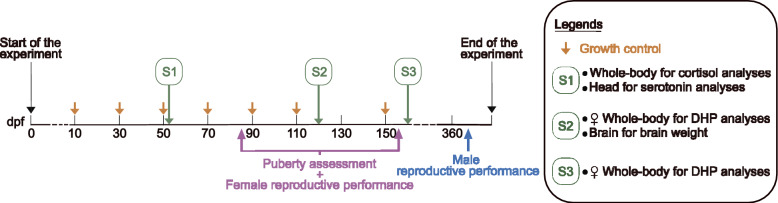
Experimental timeline. The “S” abbreviation is used for “sampling”. Time is expressed in days post-fertilisation (dpf)

Growth control experiments were performed every 20 days from 10 to 110 dpf. A final checkpoint was added at 150 dpf (Fig. 1).

Three sampling times were performed to measure hormone levels (Fig. 1): (1) S1 (52 dpf), which consisted of collecting the whole body to estimate basal cortisol levels and the head to estimate serotonin levels; and (2) S2 (100 dpf) and S3 (157 dpf), which consisted of female whole-body sampling to estimate 17α,20β dihydroxyprogesterone (DHP) levels corresponding to the maturation-inducing steroid in most female fish species. At S2, the brain was sampled to assess its weight in both males and females.

At the timepoint S1, fish are considered as juvenile, following Parichy et al. definition (i.e. “state at which most adult characteristics have been acquired in the absence of sexual maturity” associated with complete squamation and “complete loss of the larval fin fold”) [24]. Fish are considered as adult if they have produced viable gametes [24] (commonly around 3-month-old, i.e. around 90 dpf) [5]. Consequently, at S2 and S3 samplings, fish are defined as “young adult” and “more advanced adults”, respectively.

To determine whether the females were mature, reproduction tests were performed from 86 to 168 dpf. These tests are designated “puberty assessments” in the Fig. 1.

Finally, both male and female reproductive performance were tested (Fig. 1). Female performance was tested concomitantly with the puberty assessment and consisted of absolute fecundity and fertilisation rate measurements. Male performance was tested by sperm analysis at 367 dpf.

### Morphology

Four fish per replicate, for a total of 16 fish per line, were anaesthetized with 50 mg/L (10 dpf), 60 mg/L (30 dpf to 70 dpf) or 150 mg/L (from 90 to 150 dpf) of ethyl 3-aminobenzoate methanesulfonate solution (tricaine, Sigma) and NaHCO_3_ (at the same concentration as tricaine). The fish were placed under a binocular loupe (SZX7, Olympus), and pictures were taken with a camera (Cam SC50, Olympus) using specific software (CellSens CS-EN-V3, Olympus). Depending on the size of the individual, a × 0.5 or × 1.25 lens was used combined with a zoom in the range of 0.5 to 2. At 10 dpf, the pictures were taken dorsally, while they were taken laterally after 30 dpf. All the measurements were conducted using Parichy et al*.* [24] as a reference for the definition of the measured traits (standard length (SL), total length (TL), snout-vent length (SVL), snout-operculum length (SOL), height at the nape (HAN), height at the anterior of the anal fin (HAA), and eye diameter (ED)). MesurimPro software was used to perform the various measurements.

The fish were weighed at each sampling time (S1 to S3). After being euthanised by an overdose of anaesthetic (300 mg/L + 300 mg/L NaHCO_3_), the fish were wiped on absorbent paper to remove external water and weighed with a precision of ± 1 mg (Practum313-1S, Sartorius). The number of individuals weighed at 52 dpf was 32/line and varied from 10 to 24 at 100 dpf and from 5 to 15 at 157 dpf, depending on the line and sex.

At 100 dpf, after the total weight was recorded, the whole brain without the spinal cord was carefully removed and weighed. Brain weight was normalised to body weight. The number of individuals used varied between 14 and 16, depending on the line.

Additionally, global morphology analyses were performed using 150 dpf images. The software tpsDig (v2.32) was used to locate 26 landmarks (supplementary data S2), and MorphoJ (v1.07) was used for the procrustean analysis.

### Whole-body, head sampling and ELISA analyses

Immediately after death, the whole body or the head (depending on the sample taken) was wiped on absorbent paper to remove external water, weighed and placed in a tube with 10 µL of 1X phosphate-buffered saline (PBS) for 1 mg of tissue and zirconium oxide beads (ZrOB10, Next Advance). The samples were homogenised and centrifuged (5000 × *g*, 5 min, 4 °C), and the supernatants were collected and stored at − 20 °C until enzyme-linked immunosorbent assay (ELISA, detailed information in Table 1).

**Table 1:** ELISA kit information

Test	Reference	Volume of sample used	Dilution	Sensitivity	Inter-assay CV	Intra-assay CV
Serotonin	MyBiosource MBS288208	50 µL	× 7.5	< 0.35 ng/mL	16.2%	4.3% and 4.5%
Cortisol	MyBiosource MBS1608240	50 µL	No	0.021 ng/ml	17.9%	6.9% and 9.3%
DHP	MyBiosource MBS2602842	100 µL	No	< 0.06 ng/mL	48.2% (100 dpf)^*^ 29.4% (157dpf)	6.6% and 7.3% (100dpf) 3.7% and 5.0% (157 dpf)

For the serotonin and DHP tests, the standard curve was diluted in the same PBS (1 ×) as the homogenate. *the inter-assay CV was high because DHP levels were close to the detection limit of the kit

### Puberty assessment and fecundity

To determine when the fish were mature, 8 randomly chosen pairs for each line were subjected to reproduction conditions twice per week. Before dusk, one male and one female were placed in 1 L breeding tanks with a separator between them and with a grid preventing the parents from having access to the eggs. At dawn, the separator was removed. Two hours after removal of the separator, the presence (or absence) of eggs was observed.

The first trial took place at 86 dpf, and the last one occurred at 168 dpf. For a given line, the maturity onset was defined by the occurrence of the first spawning. Reproduction was considered successful when the female had spawned, regardless of whether the eggs were fertilised. After each reproductive event, the eggs were collected using an 80 µm sieve and rinsed with embryo medium (see section “Experimental animals and rearing conditions”). Each spawn was put into a Petri dish with 35 mL of embryo medium and placed in an incubator at 28.5 °C. Six hours post-fertilisation, unfertilised eggs were counted and removed using a binocular loupe. Fertilised eggs were counted to determine the absolute fecundity (i.e., total number of eggs per female = fertilised + unfertilised eggs + empty chorion) and fertilisation rate (i.e., (number of fertilised eggs/total number of eggs) × 100). The spawns were returned to 28.5 °C in an incubator and monitored every day. Dead embryos (those whose heartbeat stopped) were removed every day until hatching.

### Sperm analysis

A sperm analysis was performed at 367 dpf. After anaesthesia (150 mg/L tricaine, Sigma + 150 mg/L NaHCO_3_), the fish were gently blotted dry to remove water excess from the area around the vent and stripped to collect the sperm (n = 4 or 8, depending on the line). The volume collected varied from 0.5 to 2 µL depending on the individual. Immediately after collection, the sperm were placed in 24 µL of immobilizing solution (1 L of distilled water: 8 g of NaCl, 0.4 g of KCl, 0.16 g of CaCl_2_, 2H_2_O, 0.2 g of MgSO_4_, 7H_2_O, 0.06 g of Na_2_HPO_4_, 0.06 g of KH_2_PO_4_, 0.35 g of NaHCO_3_, and 1 g of C_6_H_12_O_6_, pH = 7.5) and kept on ice until analysis (4 h after stripping at most).

Analyses were performed using computer-assisted sperm analysis (SCA® Veterinary, Motility and Concentration, Microptic) and a microscope (Ci-L, Nikon) with a × 10 phase contrast lens and heating plate (PE120, Linkam). The sperm were activated with tap water at a ratio of 4:16, and 4 µL was immediately placed into the counting chamber (MOT-20–6 SCA^®^, Microptic) on a heating plate at 28 °C. The samples were automatically read 7 to 11 s after activation, depending on the sample.

### Statistical analyses

Statistical analyses were performed with R (v. 4.2.0). Normality and homogeneity of variance were tested using the Shapiro‒Wilk and Levene tests, respectively. When both assumptions were met, ANOVA was performed. If normality and/or homogeneity were not met, a Kruskal‒Wallis test was performed. If only homogeneity was absent, a Welch’s ANOVA was performed. If necessary, a post hoc test was conducted with a Bonferroni correction (Tukey test following ANOVA and Dunn test following the Kruskal‒Wallis test).

## Results

### Morphology

**Fig. 2:**
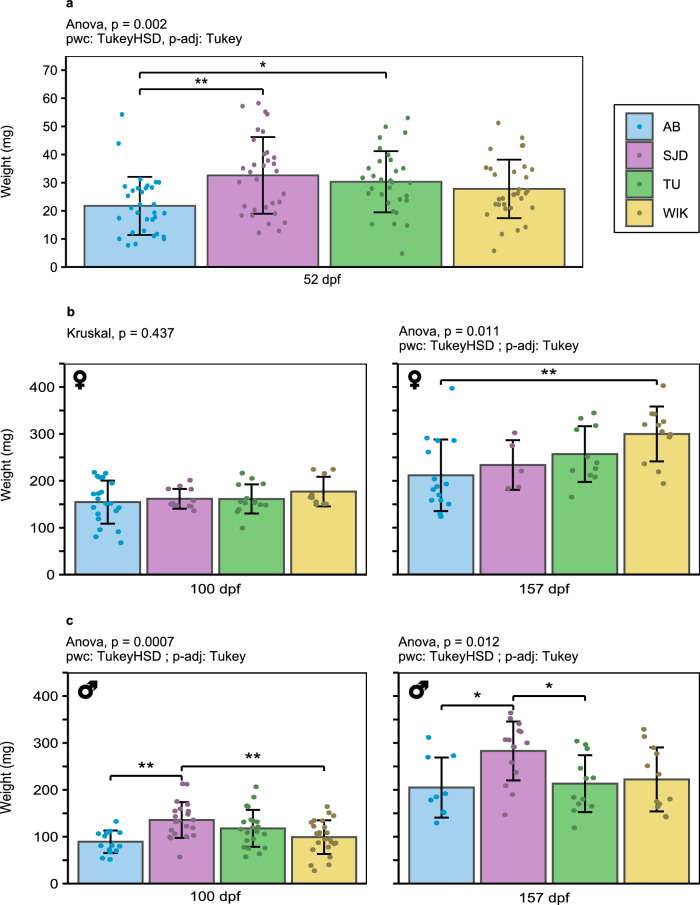
Zebrafish weight from S1 to S3. **a** Zebrafish weight at 52 dpf, undifferentiated sex; **b** Female weight at 100 and 157 dpf; **c** Male weight at 100 and 157 dpf. At 52 dpf: n = 32/line; at 100 dpf: 10 < n < 21 for females and 15 < n < 24 for males depending on the line; at 157 dpf: 5 < n < 15 for females and 9 < n < 15 for males depending on the line. The data are presented as the mean ± standard deviation. Significant differences are represented by p < 0.05 (*), p < 0.01 (**), and p < 0.001 (***)

**Fig. 3:**
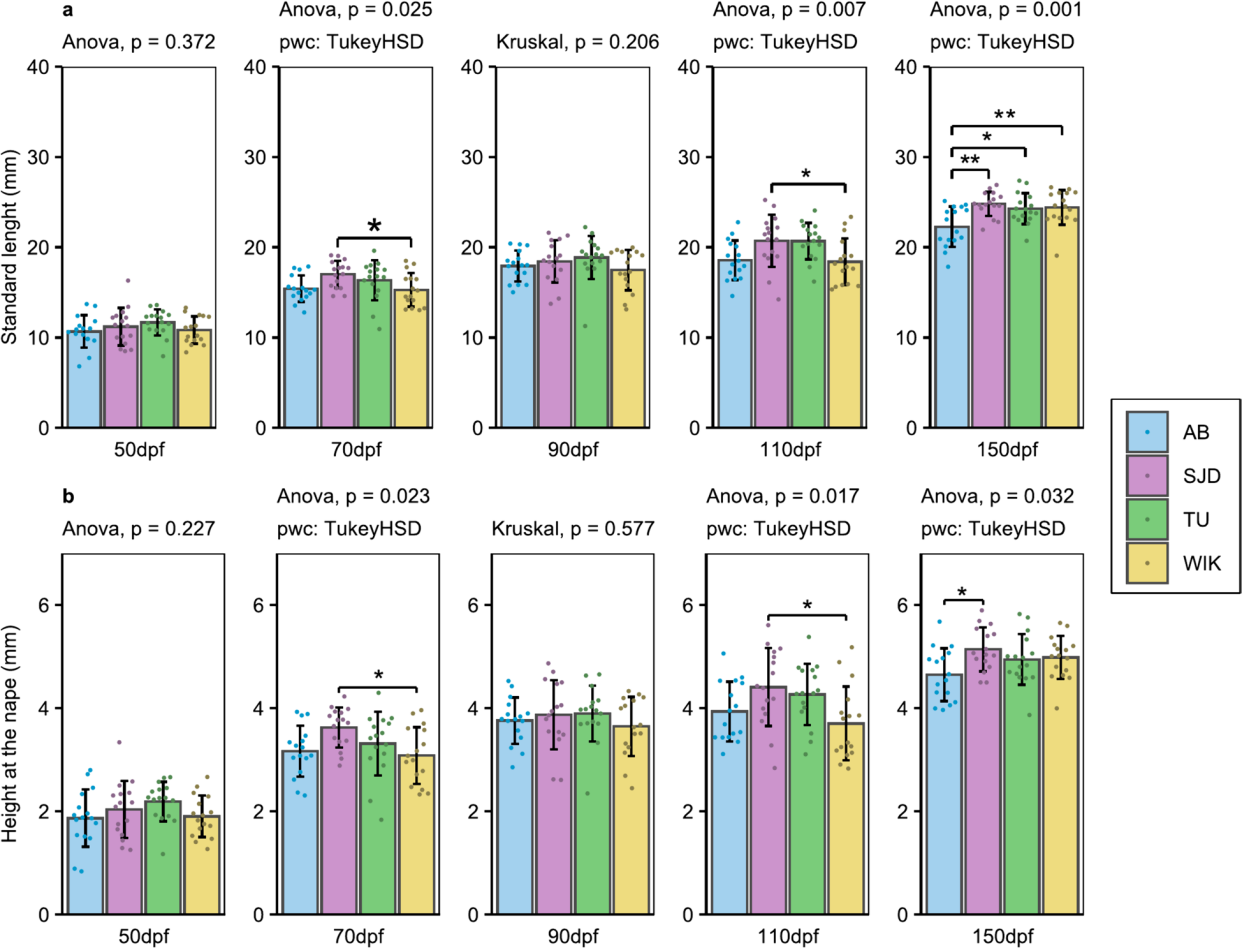
Traits measured during growth control from 50 to 150 dpf. **a** Standard length; **b** Height at the anterior of the anal fin. All the traits are in mm. n = 16/line/time. The data are presented as the mean ± standard deviation. Significant differences are represented by p < 0.05 (*), p < 0.01 (**) and p < 0.001 (***)

At the juvenile stage (timepoint S1, 52 dpf), before any clear sex identification, the weight of the AB line individuals (21.8 ± 10.3 mg) was significantly lower than that of the SJD (32.6 ± 13.6 mg, p = 0.00123) and TU (30.3 ± 1 0.9 mg, p = 0.01660) line individuals (Fig. 2a). In contrast, no obvious difference in standard length of individuals was detected up to this stage (Fig. 3a). During the remaining juvenile phase (up to 90 dpf), significant transient differences were observed among the lines at a certain time but disappeared at the next time point. This was the case for all the parameters measured, except height at the nape (HAN) and the snout-vent distance (SV) (Fig. 3b and Additional file 1).

In young adults (timepoint S2, 100 dpf, shortly after the first spawning occurrence), SJD males exhibited higher weight (135.62 ± 38.18 mg) than AB males (89.33 ± 24.05 mg, p = 0.00141) and WIK males (99.17 ± 35.91 mg, p = 0.00541) at 100 dpf (Fig. 2c). Nevertheless, only a slightly significantly greater standard length (SL) than that of WIK (18.41 ± 2.57 mm vs. 20.72 ± 2.89 mm, p = 0.0457) was detected at 110 dpf (Fig. 3a) for all the sexes combined. There was no difference in weight among females at this stage (Fig. 2b).

Later, at the more advanced adult stage (timepoint S3, 157 dpf), AB individuals had a significantly lower weight than the other lines for both sexes. More specifically, AB females had significantly lower weight than did WIK females (211.87 ± 76.36 mg vs. 299.92 ± 58.49 mg, p = 0.00647) (Fig. 2b) and AB males than SJD males (204.89 ± 64.11 mg vs. 282.93 ± 62.74 mg, p = 0.0289) (Fig. 2c). SJD males also had significantly higher weights than TU males (213.25 ± 60.68 mg, p = 0.0353) (Fig. 2c). The tendency of the AB line to be smaller than all the other lines was also confirmed by a significantly smaller SL at 150 dpf (p = 0.00135, 0.0152 and 0.00851 for the SJD, TU and WIK lines, respectively) (Fig. 3a) and to have a shorter height at the anterior of the anal fin (HAA) than the SJD line (4.65 ± 0.51 mm vs. 5.14 ± 0.42 mm, p = 0.02) (Fig. 3b). However, for global morphology, no difference was detected among any of the lines (Additional file 2).

**Fig. 4:**
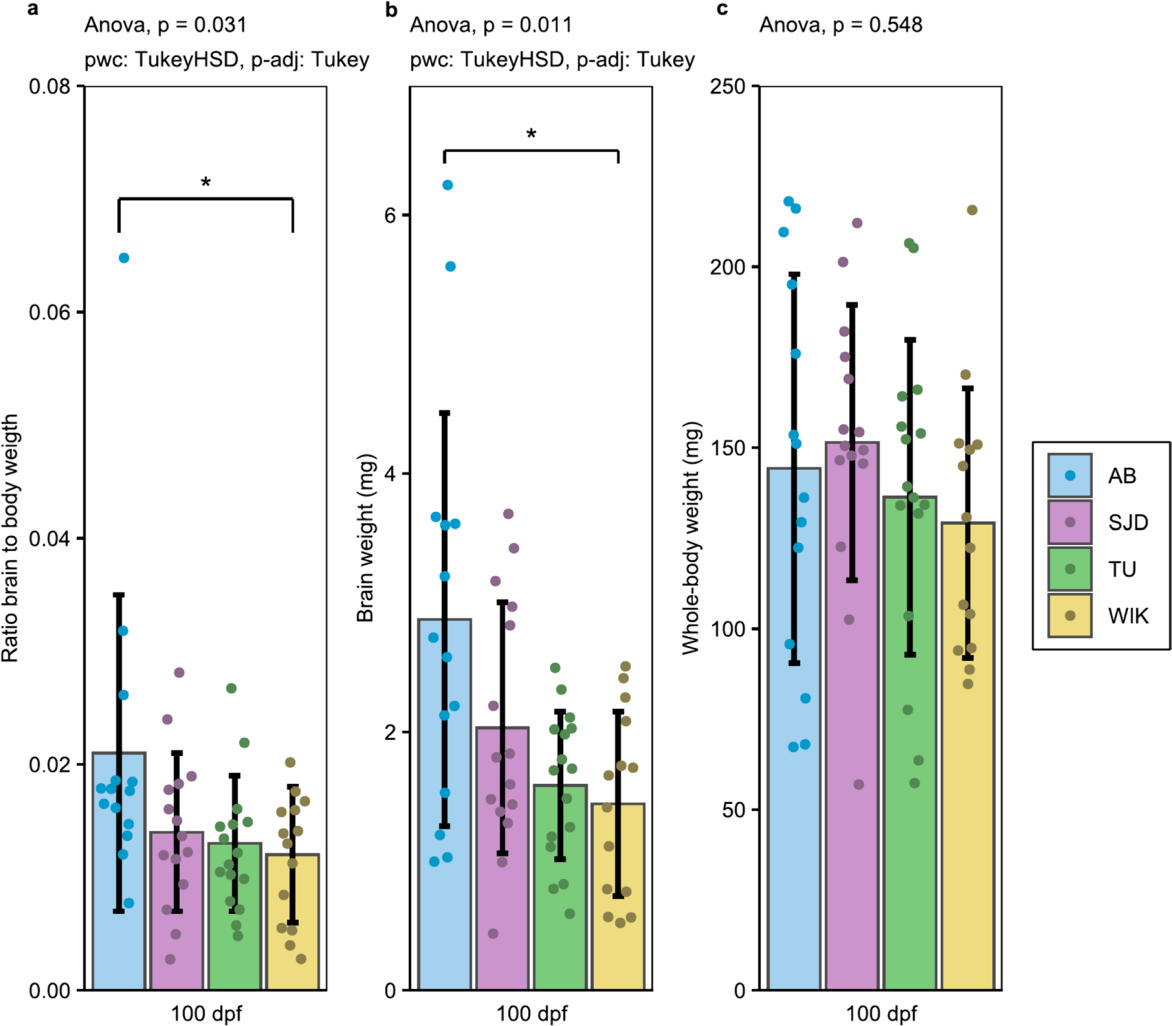
Relative and absolute brain weight at 100 dpf. **a** Ratio of brain to body weight; **b** Absolute brain weight (mg); **c** Whole-body weight of the individuals (mg). n = 14 for AB and WIK, n = 15 for SJD and n = 16 for TU***.*** The data are presented as the mean ± standard deviation. Significant differences are represented by p < 0.05 (*), p < 0.01 (**) and p < 0.001 (***)

Brain weight normalised to body weight showed very similar values among the SJD, TU and WIK lines, with averages ± standard deviations of 0.014 ± 0.007, 0.013 ± 0.006 and 0.012 ± 0.006, respectively (Fig. 4). Significant differences in absolute and relative brain weight were detected between AB and WIK lines (Fig. 4).

### Stress markers

**Fig. 5:**
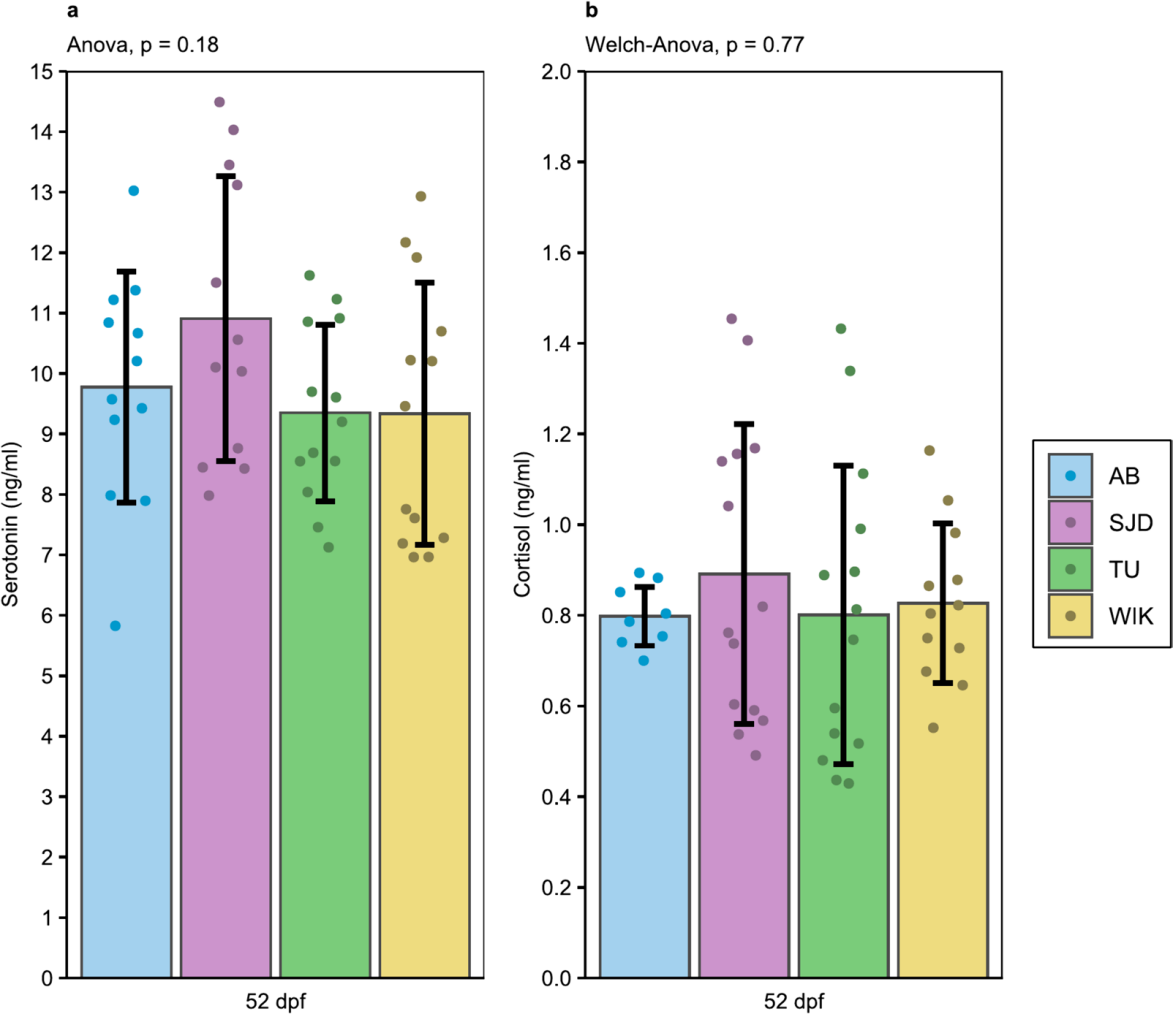
Stress markers basal levels at 52 dpf. **a** Serotonin level in head (ng/ml). n = 12 for AB and SJD, n = 13 for TU and WIK; **b** Whole-body cortisol (ng/ml). n = 8 for AB, n = 14 for SJD and TU, n = 12 for WIK. The data are presented as the mean ± standard deviation

Serotonin levels at 52 dpf were similar among the lines, ranging from 9.35 to 10.91 ng/mL. (Fig. 5a).

This similarity in average hormone levels was even more obvious for cortisol, with levels ranging from 0.80 to 0.89 ng/mL (Fig. 5b). While intra-line serotonin variations were relatively similar, we observed a great intra-line difference particularly between AB (CV = 8.2%) and TU (41.0%) for cortisol.

### Puberty

**Fig. 6:**
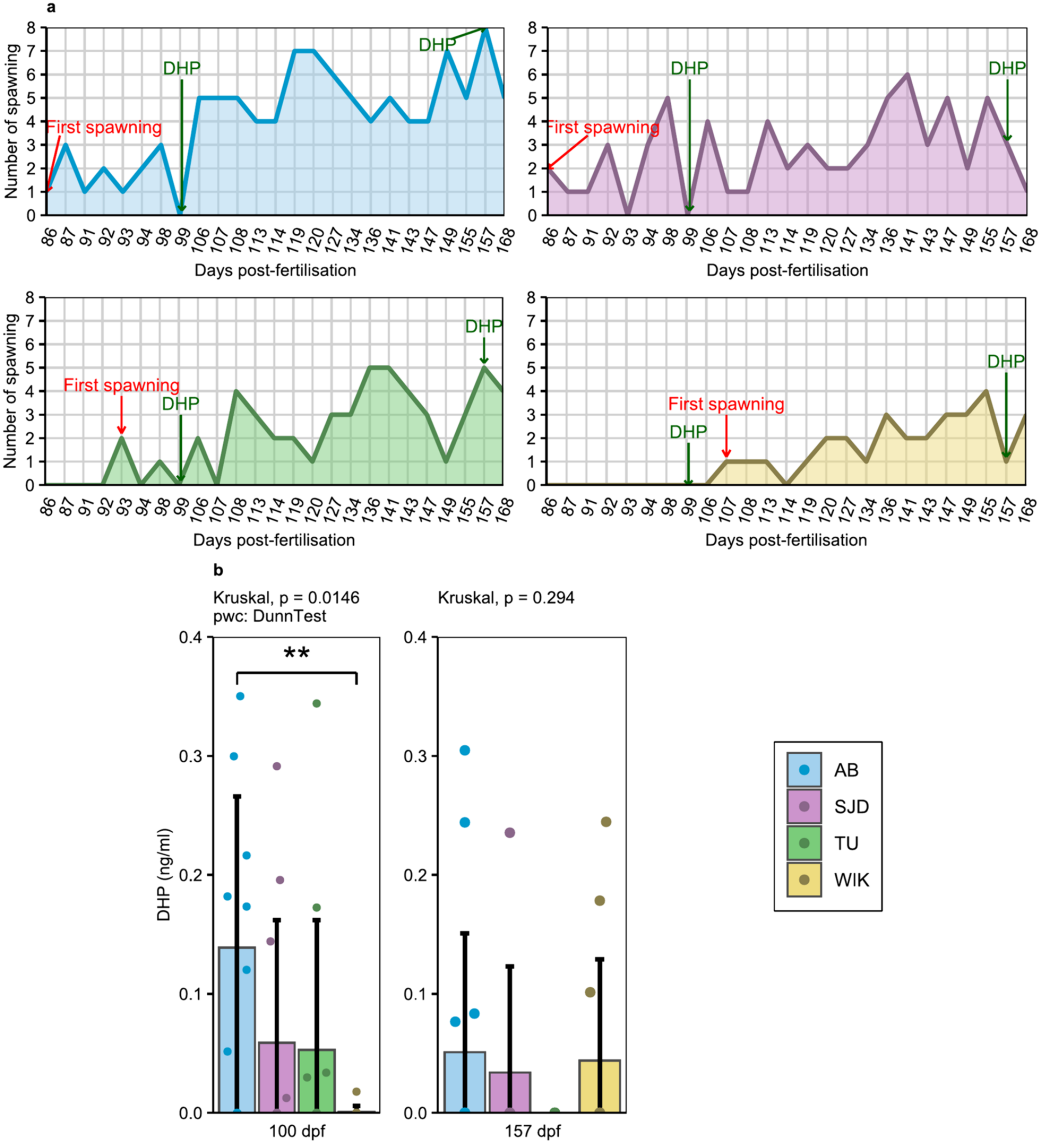
Puberty assessment. **a** Daily number of spawn for each line throughout the tested period (from 86 to 168 dpf) (n = 8 couples/line each time) and (**b**) whole-body DHP at 100 dpf (n = 10 for the AB line, n = 11 for the SJD and TU lines, n = 12 for the WIK line) and 157 dpf (n = 14 for the AB line, n = 7 for the SJD line, n = 11 for TU and n = 12 for the WIK line). The data shown represent the mean ± standard deviation

The AB and SJD females were the first to spawn at 86 dpf. The first spawning in the TU line occurred 7 days later, at 93 dpf (Fig. 6a). At this stage, only the WIK line had not yet spawned, and this delay in puberty was confirmed at 100 dpf by analysis of female whole-body DHP (17α,20β dihydroxyprogesterone) levels (Fig. 6b). Seventy percent of the AB individuals produced detectable levels of DHP, with a mean of 0.14 ± 0.13 ng/mL. The percentage of individuals producing detectable amounts of DHP was the same for the SJD and TU lines (i.e., 36.4%), with average levels of 0.06 ± 0.10 ng/mL and 0.05 ± 0.11 ng/mL, respectively. Among all the lines, WIK showed the lowest quantity of DHP produced, as only one individual had detectable DHP (i.e., 8.3%) (average level of 0.001 ± 0.005 ng/mL). Moreover, DHP levels were significantly lower in WIK females than in AB females (p = 0.00715) (Fig. 6b).

The WIK line spawned for the first time at 107 dpf, more than 20 days after the AB and SJD lines (Fig. 6a). Moreover, no significant differences were detected among lines in whole-body DHP female samples collected at 157 dpf, although none of the TU individuals tested produced DHP at this time (Fig. 6b).

Importantly, regardless of the line or sampling time, DHP was never detectable in 100% of the tested individuals.

### Reproductive performance

**Fig. 7:**
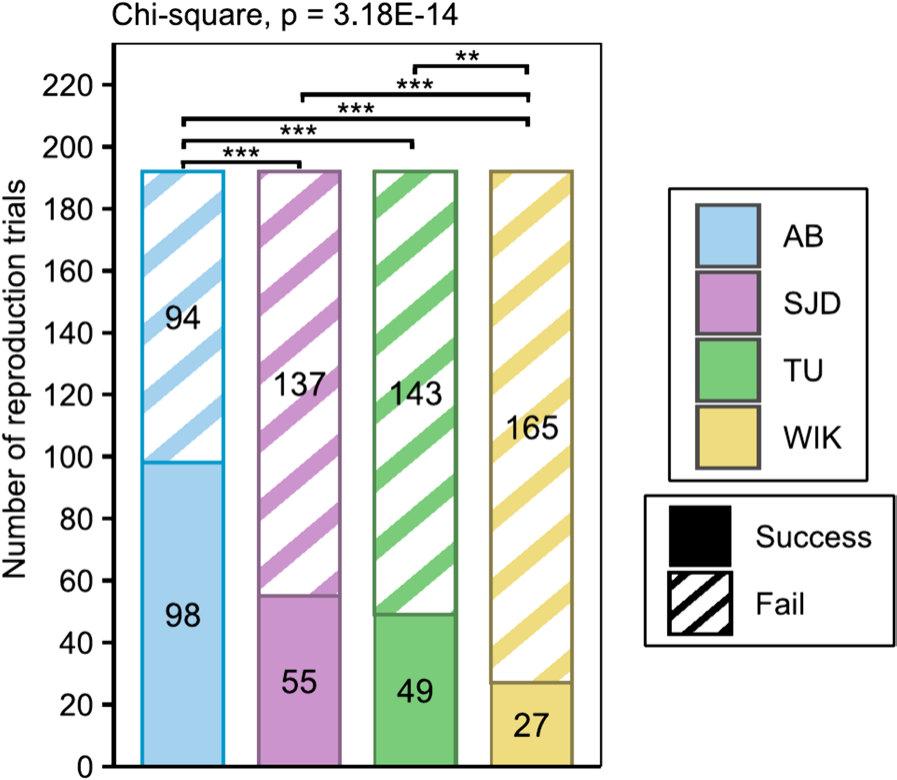
Total number of reproductive successes and failures per line throughout the test period. Reproduction was considered successful when the female had spawned, regardless of whether the eggs were fertilised. n = 8 couples/line/day. A chi-square test was used to determine whether spawning frequencies differed among lines. Significant differences are represented by p < 0.05 (*), p < 0.01 (**) and p < 0.001 (***)

Over the whole period tested, the AB line showed a significantly higher number of successful spawnings (51.04%) (i.e., spawning events, regardless of whether the eggs were fertilised) than did the other lines (28.64%, 25.52% and 14.06% for SJD, TU and WIK, respectively, 2.47 × 10^–14^ < p < 1.20 × 10^–5^). In contrast, WIK females had the lowest ratio of successful spawning (14,06%) among all lines (2.47 × 10^–14^ < p < 7.15 × 10^–3^) (Fig. 7).

**Fig. 8:**
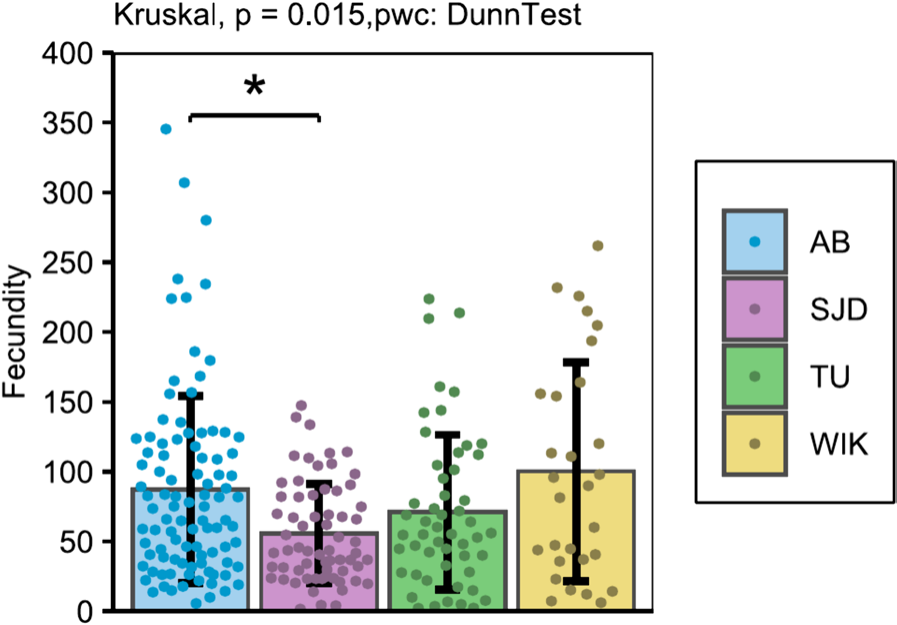
Absolute fecundity for each line throughout the test period. The data shown represent the mean ± standard deviation. Significant differences are represented by p < 0.05 (*), p < 0.01 (**) and p < 0.001 (***)

The mean number of eggs released (fertilised or not) by AB females was significantly higher than that released by SJD females (p = 0.0221) (Fig. 8).

**Fig. 9:**
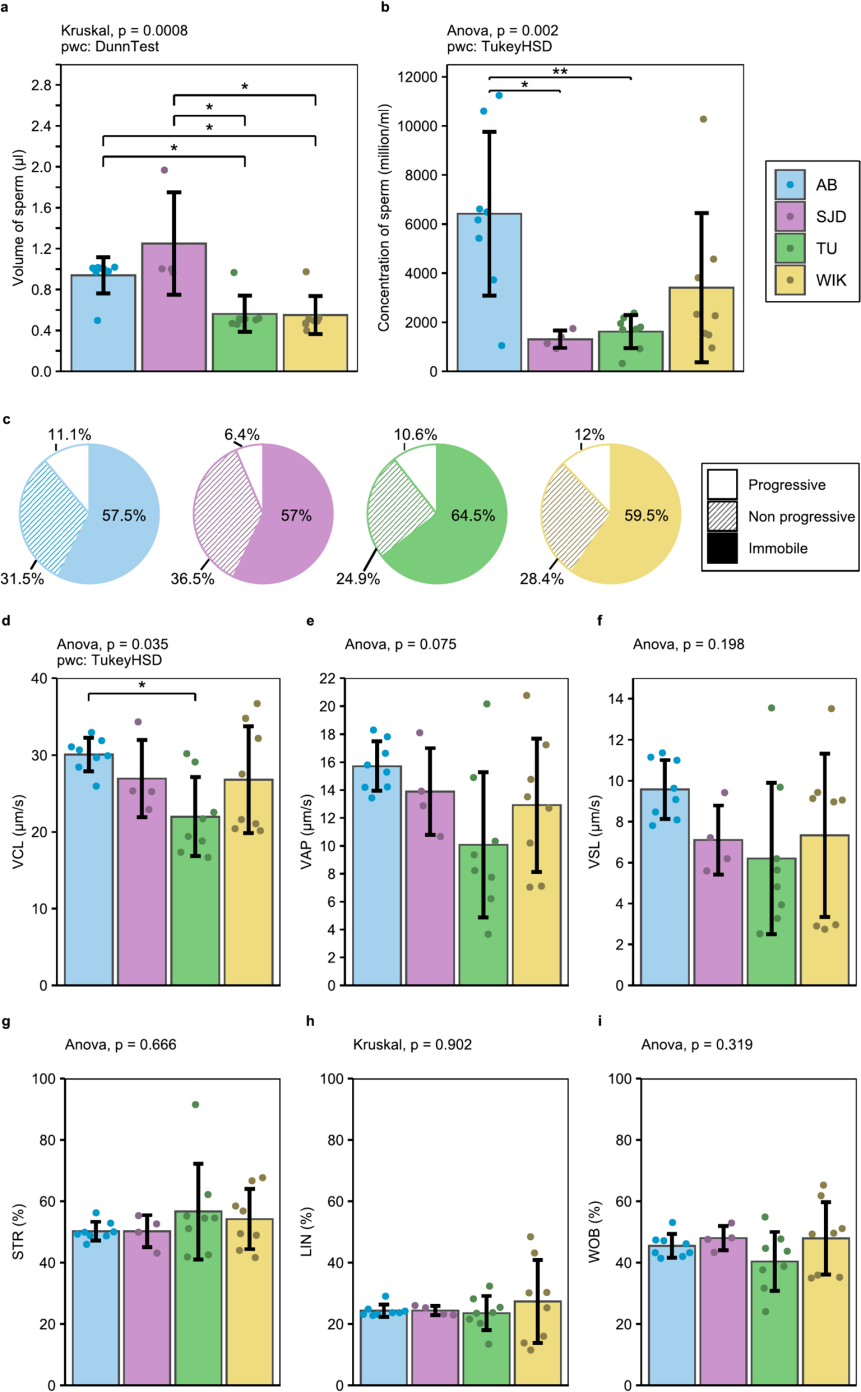
Sperm parameters analysed by CASA. **a** Volume (µl) of sperm collected; **b** Concentration (million/ml) of spermatozoa; **c** Proportion (%) of progressive (dot pattern), non-progressive (stripe pattern) and immobile (plain pattern) spermatozoa; **d** VCL—Curvilinear velocity (µm/s) for all motile spermatozoa; **e** VAP—Average path velocity (µm/s) for all motile spermatozoa; **f** VSL—Straight-line velocity (µm/s) for all motile spermatozoa; **g** STR- Straightness (%) for all motile spermatozoa; **h** LIN—linearity (%) for all motile spermatozoa; **i** WOB—wobble (%) for all motile spermatozoa. n = 8 for AB, TU and WIK, n = 4 for SJD. The data shown represent the mean ± standard deviation. Significant differences are represented by p < 0.05 (*) and p < 0.01 (**)

**Fig. 10:**
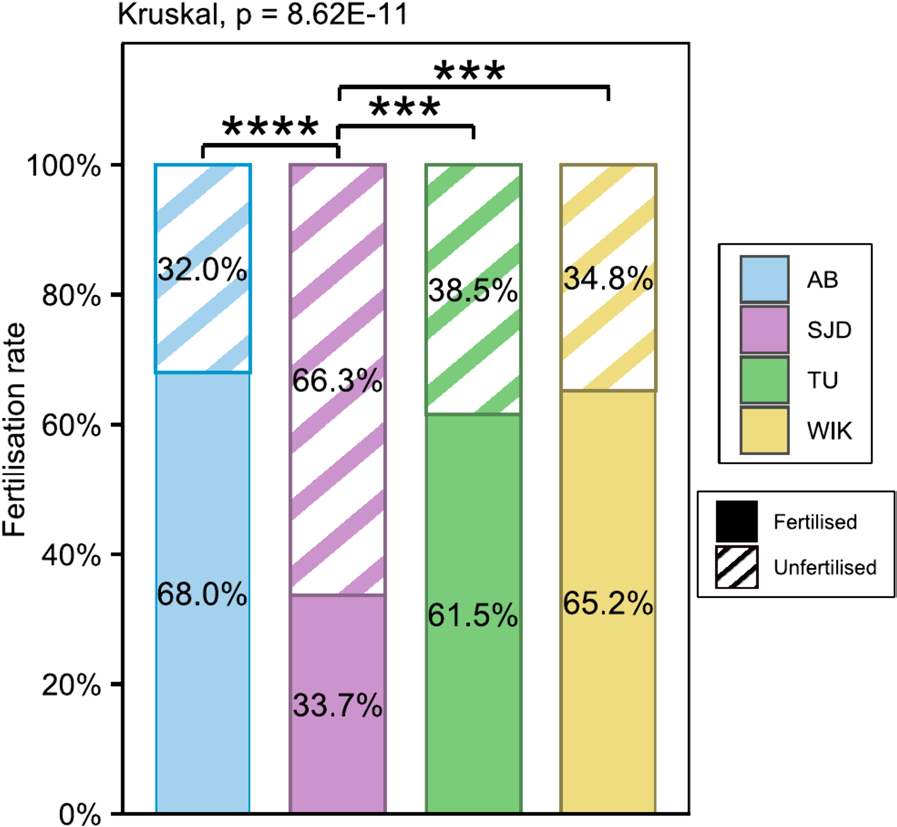
Fertilisation rate for each line throughout the tested period. The data shown represent the means. Significant differences are represented by p < 0.05 (*), p < 0.01 (**) and p < 0.001 (***)

Among males, the AB and SJD lines produced significantly greater volumes of sperm than the TU and WIK lines (p = 0.0476 and 0.0217 for AB vs. TU and WIK, respectively; p = 0.0231 and 0.0117 for SJD vs. TU and WIK, respectively) (Fig. 9a). Compared with the SJD and TU males, the AB males exhibited much more concentrated sperm (p = 0.0118 and 0.00353, respectively) (Fig. 9b). No significant difference was observed in the proportion of progressive, non-progressive or immobile spermatozoa among any of the 4 lines (Fig. 9c). Concerning motility measurements, only the curvilinear velocity (VCL) significantly differed between the AB and TU lines (p = 0.0207), considering all the motile spermatozoa (Fig. 9d, e, f, g, h, i). For rapidly progressing spermatozoa only, the AB line exhibited significantly greater average path velocity (VAP) and linear velocity (VSL) than did the SJD line (p = 0.0482 and 0.0485, respectively) (Additional file 3). Furthermore, the fertilisation rate in the SJD line was lower than that in all the other lines (p = 3.8 × 10^–11^, 1.21 × 10^–5^ and 5.96 × 10^–4^ for the AB, TU and WIK lines, respectively) (Fig. 10).

## Discussion

In recent decades, the zebrafish model has become increasingly popular in research [22, 23]. Consequently, to meet the needs of researchers, several zebrafish lines have been made available [6]. Each of these lines has evolved through a different process of adaptation to laboratory conditions, marked by distinct and specific choices of the founder populations, the genetic selection methods applied, the way the lines were reproduced and the number of generations reared in captivity [5, 7, 18–20]. Taken together, these findings may explain the differences in phenotypes among these lines. The aim of this study was to compare key parameters related to growth, stress response, and reproduction in commonly used WT lines bred under the same laboratory conditions to identify potential differences and similarities among them.

Stress hormones are classical physiological indicators used to evaluate stress responses in fish. In this study, the cortisol levels were very similar among the four tested lines. These measured whole-body cortisol levels can be interpreted as the basal cortisol level since the hormone peak in zebrafish occurs 15 min after stress induction [25, 26], while levels were measured less than 5 min after capture in this study. Similarly, equivalent serotonin brain levels between the AB and TU lines have been reported in the literature [27], and we confirmed this trend among the four lines tested (Fig. 5). This similarity between the two hormones might be explained by the relationship between serotonin and cortisol, the former playing a role in the release of the latter [28]. Therefore, these results are an initial indication that the level of stress hormones in zebrafish is potentially robust to the different domestication routes applied and that the line choice is not a key parameter impacting the results for endocrine evaluation of basal stress in zebrafish.

One of the factors that makes the zebrafish an increasingly popular animal model is its short life cycle. Zebrafish are mature at approximatively 3 months of age [5], even if puberty is more related to body growth than to age in both sexes [29, 30]. Our comparisons of the AB, SJD, TU and WIK lines highlighted differences in both growth and puberty timing. The AB and SJD lines were mature at 86 dpf, while TU was mature at 93 dpf and WIK at 107 dpf (Fig. 6), but no difference in weight was found at 100 dpf (Fig. 2B) between females. Regarding the size at both measurement points closest to the time of puberty for each line, no difference in standard length or height at nape was detected at 90 dpf (Fig. 3). No difference in total length was detected at 90 dpf or 110 dpf (Fig. 3), which was the point closest to the time of puberty for each line. At this stage, all females weighed more than 150 mg with a mean total body length of 2.3 cm, regardless of the line, while the literature reports that female puberty is initiated after the threshold of 100 mg in weight and a total length of 1.8 cm [29]. However, this latter threshold was determined using the albino zebrafish line [29], and those data were not confirmed for other lines. Here, we demonstrate that at the same weight and total length, not all lines had mature females at the same time, highlighting the fact that the initiation of puberty differs among zebrafish lines and may be disconnected from fish growth. Nonetheless, we must remain cautious about these results because different individuals were used for puberty evaluation and DHP (17α,20β dihydroxyprogesterone) level studies, the maturation-inducing hormone in zebrafish [31]. In addition, although the samples were all taken at the same time, importantly, DHP levels follow a daily cycle marked by a peak at the time of ovulation in the ovaries [32]. Given that few individuals produced DHP, it is possible that the production peak had already passed (if the females had already spawned) by the time the sample was collected. Differences in the age at which puberty is reached among populations of the same species maintained under similar breeding conditions have already been demonstrated in cod (*Gadus morhua*) [33]. This highlights that the induction of puberty in female fish raised under similar conditions can vary depending on the line and its domestication history. In our case, the data suggest that the choice of zebrafish lines with precocious puberty (AB or SJD lines) is relevant for accelerating the obtention of successive generations.

Although the AB and SJD females matured synchronously and precociously, the AB line showed better reproductive performance than the other lines, including SJD, as indicated by the AB line having the highest spawn success rate, fertilization rate and fecundity. A similar conclusion may be drawn for males whose sperm quality (volume, spermatocrit and spermatozoa speed) was better in the AB line than in the other lines. These results might be explained by the fact that AB is the oldest line to have been established. Indeed, comparable results have already been demonstrated in Nile tilapia (*Oreochromis niloticus*, L.), for which females from two lines domesticated for several years under similar conditions exhibited better reproductive performance than females from two other lines domesticated more recently [34]. It is plausible that the AB line has acquired a genetic stability and adaptive traits over time that have not yet been achieved in the other lines, which could explain its superior reproductive performance.

Regarding the general morphology, the traits measured in this study are those described by Parichy et al*.* as characteristics that undergo sufficient changes during postembryonic development to enable the assessment of developmental progression [24]. All measured traits significantly differed at least once over the 7 measurement times. However, the differences tended to disappear at the subsequent time point, suggesting that these inter-line specificities are transitory, as previously demonstrated in other species, such as cod [33]. Interestingly, the adults of the AB were smaller than the adults of all the other lines. This finding complements the findings of previous research, which focused on growth during postembryonic periods. The AB and TU lines exhibited no disparities in terms of SL before the adult stage (7 to 87 dpf) [27]. Similarly lack of difference was observed between the TU line and the WIK line regarding SL and HAA (5 to 40 dpf) [35]. However, the latter study reveals that while there is no disparity in growth, certain developmental milestones can be achieved at distinct SL between lines, underscoring the importance of employing named stages rather than age to describe developmental evolution [35]. The AB line also showed the heaviest absolute and relative brain weight, with a significant difference from that of the WIK line. Such differences between lines of model species have already been demonstrated in rats, where the brains of Wistar lines are larger than those of Long–Evans lines, in addition to there being anatomical differences between the two lines, suggesting better performance of the Long–Evans line in motor tasks [36]. This is also the case in other fish models such as medaka, where differences have been highlighted not only in the total volume of the brain but also in brain morphology [37]. Domestication, which is the adaptation of a species to captivity, tends to reduce the brain size of domesticated animals compared with that of their wild counterparts. This phenomenon has been shown in multiple species, including lab species such as rats [38], as well as in many fishes such as guppies (*Poecilia reticulata*), where females of the first generation had smaller brains than did those of a wild-caught population [39], salmon (*Oncorhynchus tshawyscha*) [40] and rainbow trout (*Oncorhynchus mykiss*) [41]. In all these examples, changes are rapid, occurring within the first generations of domestication. In the present zebrafish case, the line domesticated for the longest period (i.e., AB) showed a higher brain weight normalised to body weight than a more recent line (i.e., WIK) (although the exact number of generations for each is not known, approximately 25 years separate the establishment of these two lines). This result contradicts our hypothesis that the most recent line should have the largest brain or that all lines are similar (because they have been domesticated for several generations and are now stabilised). Thus, further investigations are necessary to determine more precisely which area of the brain is modified, to understand intraspecies specificities and to determine the potential impact on fish behaviour.

## Conclusion

Our results highlight for the first time that differences may exist among various wild-type zebrafish lines reared in exactly same conditions, particularly concerning both development and reproduction. These differences could be a problem for reproducibility of research results, especially because many studies do not precisely mention the zebrafish line used or the origin of the line. Opting for a specific line could influence the results of a given study and affect the generalisability of the findings to other populations. Importantly, selecting a commonly used line does not necessarily ensure the representativeness of the overall results. When selecting a zebrafish line, it is crucial to consider the biological relevance of the characteristics specific to each line. The line choice must be deliberate, guided by an in-depth understanding of the specific characteristics sought and the known genetic and/or phenotypic variations among the zebrafish lines.

## Supplementary Information

Additional file 1. Traits measured during growth control from 10 dpf to 150 dpf.Total length;Snout-Operculum distance;Height at nape;Snout-Vent distance;Eye diameter. All the traits are in mm. Data are presented as mean ± standard deviation. Significant differences are represented by: p < 0.05; p < 0.01; p < 0.001

Additional file 2. Comparison of global morphology at 150 dpf. Comparison were assessed using Discriminant Function Analysis based on the procrustean coordinates

Additional file 3. Sperm parameters analysed by CASA. *VCL* curvilinear velocity, *VAP* average path velocity, *VSL* straight-line velocity, *STR* straightness, *LIN* linearity, *WOB* wobble. One or more significant differences are indicated by the letters in brackets. A different letter between 2 values of the same parameter indicates a significant difference.

## Data Availability

All data generated or analysed during this study are included in this published article and its supplementary information files. The datasets used and/or analysed during the current study are available from the corresponding author on reasonable request.
