# Supplementary figures and images for: Comparative study of the growth, stress status and reproductive capabilities of four wild-type zebrafish (Danio rerio) lines
Source: Biol Res. 2024 Sep 19;57:67. doi: 10.1186/s40659-024-00549-3 (PMC11411999; doi:10.1186/s40659-024-00549-3)

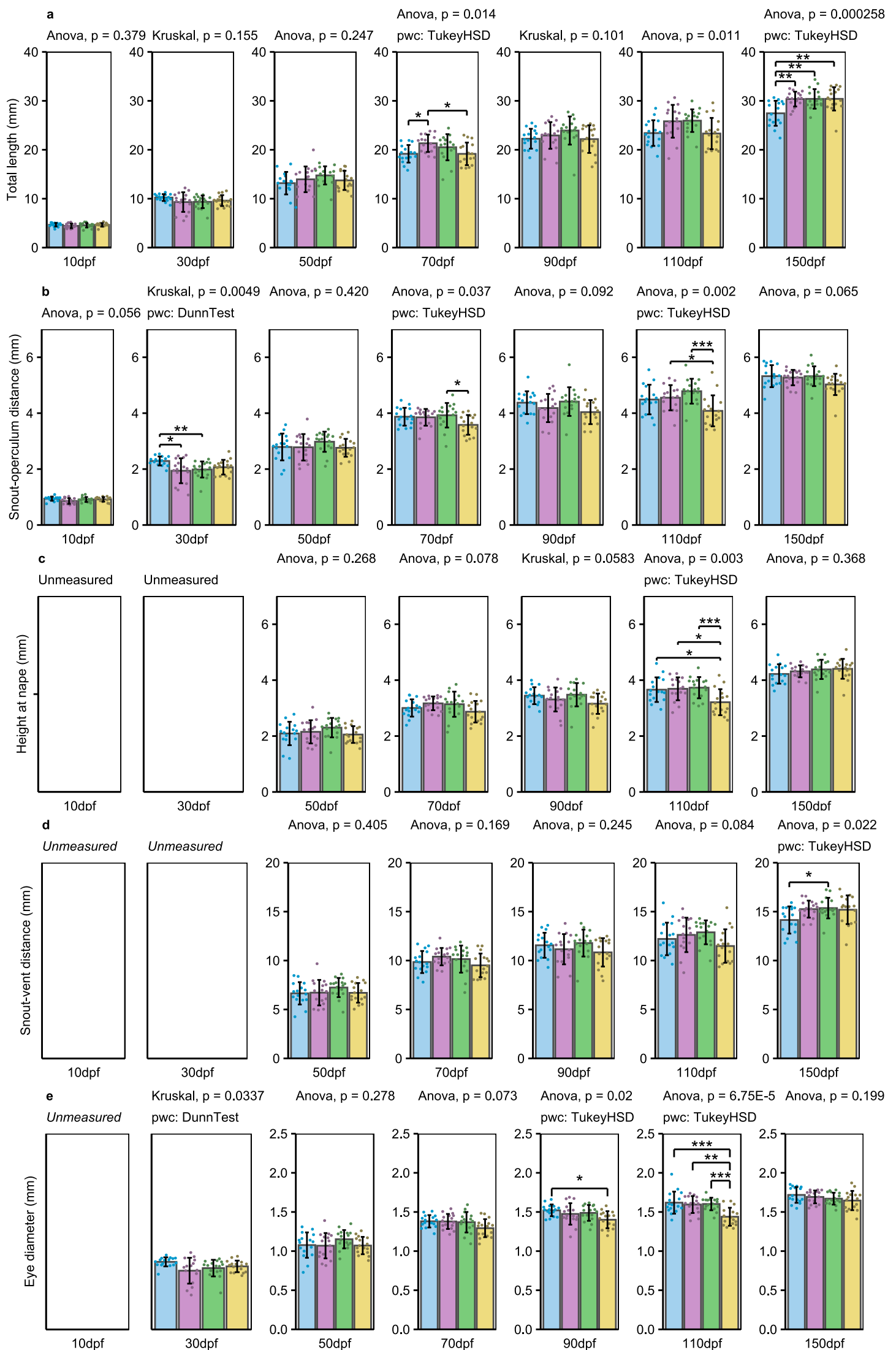

Supplement: Supplementary file 1 — Additional file 1. Traits measured during growth control from 10 dpf to 150 dpf.Total length;Snout-Operculum distance;Height at nape;Snout-Vent distance;Eye diameter. All the traits are in mm. Data are presented as mean ± standard deviation. Significant differences are represented by: p < 0.05; p < 0.01; p < 0.001 [file 40659_2024_549_MOESM1_ESM.pdf]

AB

SJD

TU

SJD

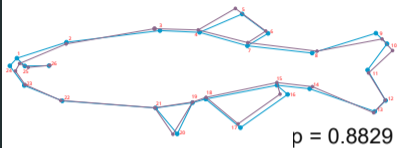

TU

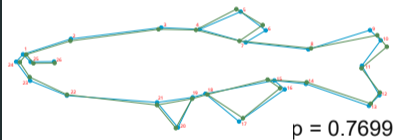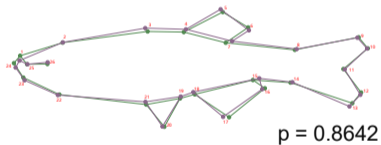

WIK

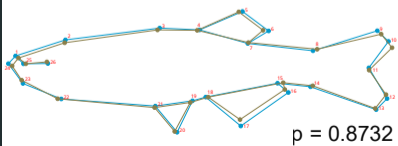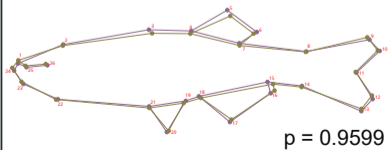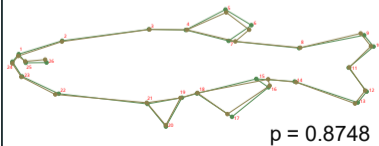

Supplement: Supplementary file 2 — Additional file 2. Comparison of global morphology at 150 dpf. Comparison were assessed using Discriminant Function Analysis based on the procrustean coordinates [file 40659_2024_549_MOESM2_ESM.pdf]
